# Investigation of mechanisms of bendiocarb resistance in *Anopheles gambiae* populations from the city of Yaoundé, Cameroon

**DOI:** 10.1186/s12936-016-1483-3

**Published:** 2016-08-22

**Authors:** Christophe Antonio-Nkondjio, Rodolphe Poupardin, Billy Fossog Tene, Edmond Kopya, Carlo Costantini, Parfait Awono-Ambene, Charles S. Wondji

**Affiliations:** 1 Laboratoire de Recherche sur le Paludisme, Organisation de Coordination pour la lutte Contre les Endémies en Afrique Centrale (OCEAC), P.O. Box 288, Yaoundé, Cameroon; 2 Faculty of Sciences, University of Yaoundé I, P.O. Box 337, Yaoundé, Cameroon; 3 Institut de Recherche pour le Développement (IRD), UR 016, 911, Avenue Agropolis, P.O. Box 64501, 34394 Montpellier Cedex 5, France; 4 Vector Group Liverpool School of Tropical Medicine Pembroke Place, Liverpool, L3 5QA UK

**Keywords:** Bendiocarb resistance, *Anopheles gambiae*, P450 monooxygenase, metabolic resistance, Yaoundé, Cameroon

## Abstract

**Background:**

Resistance to the carbamate insecticide bendiocarb is emerging in *Anopheles gambiae* populations from the city of Yaoundé in Cameroon. However, the molecular basis of this resistance remains uncharacterized. The present study objective is to investigate mechanisms promoting resistance to bendiocarb in *An. gambiae* populations from Yaoundé.

**Methods:**

The level of susceptibility of *An. gambiae s.l.* to bendiocarb 0.1 % was assessed from 2010 to 2013 using bioassays. Mosquitoes resistant to bendiocarb, unexposed and susceptible mosquitoes were screened for the presence of the Ace-1^R^ mutation using TaqMan assays. Microarray analyses were performed to assess the pattern of genes differentially expressed between resistant, unexposed and susceptible.

**Results:**

Bendiocarb resistance was more prevalent in mosquitoes originating from cultivated sites compared to those from polluted and unpolluted sites. Both *An. gambiae* and *Anopheles coluzzii* were found to display resistance to bendiocarb. No G119S mutation was detected suggesting that resistance was mainly metabolic. Microarray analysis revealed the over-expression of several cytochrome P450 s genes including *cyp6z3, cyp6z1, cyp12f2, cyp6m3* and *cyp6p4*. Gene ontology (GO) enrichment analysis supported the detoxification role of cytochrome P450 s with several GO terms associated with P450 activity significantly enriched in resistant samples. Other detoxification genes included UDP-glucosyl transferases, glutathione-S transferases and ABC transporters.

**Conclusion:**

The study highlights the probable implication of metabolic mechanisms in bendiocarb resistance in *An. gambiae* populations from Yaoundé and stresses the need for further studies leading to functional validation of detoxification genes involved in this resistance.

## Background

Malaria prevention largely relies on the use of measures, such as long-lasting insecticidal nets (LLINs) and indoor residual spraying (IRS) [1]. Of the four insecticides classes used in public health, pyrethroids are by far the most widely used [1]. During the past decades, overreliance on pyrethroids in public health and agriculture, led to rapid expansion of pyrethroid resistance in malaria vectors populations which now threatens the continued effectiveness of current control efforts [2]. Resistance to pyrethroids is mainly due to mutations in the knock down genes (*kdr*) and metabolic detoxification mechanisms and is largely prevalent in all major malaria vectors [3–5]. Because of rapid spread rate of this resistance across sub-Saharan Africa, effective measures are needed to mitigate its impact. The World Health Organization (WHO) recommends application of insecticides having different mode of action or temporal replacement by different insecticide classes in case of resistance [6]. Carbamates and organophosphates due to their different mode of action, are actually considered as suitable alternative insecticides to pyrethroids for vector control such as IRS [7–10]. Field experiments conducted across West Africa showed the effectiveness of carbamates and organophosphates against pyrethroid resistant malaria vector populations [11, 12]. There is, an increasing number of countries which have started introducing the use of carbamate in their national vector control strategy [7, 9, 10]. However, increasing reports of carbamates resistance in the main malaria vectors across sub-Saharan Africa [13–17], could jeopardize current efforts to implement appropriate resistance management strategies against malaria vectors. Despite current expansion of bendiocarb resistance little is known on mechanisms promoting this resistance in Central African mosquito populations.

In Cameroon, despite efforts made over the past years to control malaria, the disease is still considered, a major threat [18, 19]. Major vectors in the country display high level of pyrethroid resistance [20–22]. Studies undertaken in the cities of Douala and Yaoundé, reported particularly high prevalence of pyrethroid and DDT resistance in both *Anopheles gambiae* and *Anopheles coluzzii* [23–25]. The use of insecticide tools for vector control in households, the selective pressure of pollutants in breeding habitats and uncontrolled use of pesticides in small scale urban vegetable farming are all considered to have caused, the fast evolution of insecticide resistance which is now also affecting bendiocarb [23, 25, 26]. However, the molecular basis of carbamate resistance remained uncharacterized in *An. gambiae* populations in Cameroon. Such information is crucial to guide the implementation of appropriate resistance management strategies to prolong the effectiveness of carbamates in Cameroon. The main resistance mechanisms to carbamates involved metabolic resistance and target-site resistance. Metabolic resistance to carbamates is often conferred by the up-regulation of detoxification genes such as cytochrome P450 s [27, 28] or carboxylesterases [29–31].

Target-site resistance to carbamates and organophosphates is conferred by a single point mutation causing acetylcholinesterase inhibition [32, 33]. The mutation encoded by the *Ace*-*1*^*R*^ gene induces a substitution from glycine to serine at position 119 (G119S). The G119S mutation has also been recorded in several species including *Culex quinquefasciatus*, *Anopheles albimanus* and *An. gambiae* [33–38]. Recent findings reported the duplication of this mutation in some *An. gambiae* individuals [39, 40]. In Cameroon, no G119S mutation has up to now been reported in *An. gambiae* population and the underlying molecular basis of the carbamate resistance in this major malaria vector remain to be established. The present study seeks to characterize mechanisms involved in the ongoing mosquito resistance to carbamate in the city of Yaoundé. The study also traces the dynamics of *An. gambiae* susceptibility to bendiocarb between 2010 and 2013.

## Methods

### Study site

Mosquito collections were conducted in districts of the city of Yaoundé (3°51′N 11°30′E). Yaoundé the capital city of Cameroon, is situated within the Congo-Guinean phytogeographic domain and display an equatorial climate consisting of four seasons: two rainy seasons (March–June and September–November; annual rainfall 1700 mm) and two dry seasons (December–February and July–August).

### Mosquito collection

Mosquito larvae were collected at all stages in water collections across the city of Yaoundé and reared separately according to their breeding habitats characteristics classified as cultivated, polluted or non polluted sites. Water collections with organic wastes were considered as polluted, non-polluted breeding sites were water collections without any sign of organic pollution, cultivated breeding sites were water collections associated with farming practices. In the laboratory, larvae were transferred into distilled water and reared separately at room temperature. During this period, they were fed using fish food until the pupa stage. Pupa were collected in cups and placed inside cages covered with netting for emergence.

### Insecticide bioassays

Bioassays were conducted from October 2010 to December 2013 using 2–4 days old females emerging from larvae collected on the field. Morphological identification keys [41] were used to differentiate members of the *An. gambiae* complex to other mosquito species at both the larval and the adult stages. Unfed *An. gambiae s.l.* females aged 2–4 days were exposed to 0.1 % bendiocarb, 4 % DDT (dichloro-diphenyl-trichloroethane), 0.75 % permethrin, 0.05 % deltamethrin and 4 % malathion in susceptibility test kits from the WHO, following standardized procedures [42]. For bioassays using piperonyl butoxide (PBO) as synergist, unfed *An. gambiae* females were pre-exposed to 4 % PBO papers for 1 h before being immediately exposed for another 1 h to bendiocarb. Mortality was scored after 24 h but for mosquitoes surviving exposition to bendiocarb, they were maintained in observation for a total period of 48 h before storage in RNAlater. For each bioassay, exposition of mosquitoes to untreated papers was also undertaken as controls. Abbot formula [43] was used to adjust mortality rate in tested samples if the control group mortality rate was 5–20 %. WHO recommendations [42] were applied for classifying mosquitoes as resistant or susceptible.

Odd ratio calculations were undertaken to assess any association between phenotypes and genotypes [(resistants genotype A*susceptibles genotype B)/(susceptibles genotype A*resistants genotype B)]. Odd ratio estimates, mortality rates, the 95 % confidence intervals and p values were calculated with the software MedCalc V11.5.0.0.

### Molecular identification of species and genotyping of Ace-1^R^ G119S mutation

Genomic DNA utilized for the identification of *An. gambiae s.l.* species and the screening of the Ace-1^R^ G119S mutation, was extracted from a leg or wing of adult mosquitoes by the Livak technique [44]. A polymerase chain reaction (PCR) was used for *An. gambiae* species identification [45]. The presence of the G119 mutation was screened using TaqMan assays as previously described [46]. TaqMan reactions were undertaken using the Agilent MX3005P machine. Each reaction was conducted in a 10 μl final volume with 1xSensiMix (Bioline), 800 nM of each primer and 200 nM of each probe.

### Microarray experiments

Microarray experiments were conducted using only *An. gambiae* samples originating from cultivated sites where bendiocarb resistance was most prevalent. Differentially transcribed genes were compared between resistant, control (unexposed) and susceptible (Kisumu) samples.

Pools of ten mosquitoes were used for total RNA extraction with the PicoPure RNA isolation Kit (Arcturus, Applied Biosystems, Mountain View CA USA). Each sample was constituted of three biological replicates. Total RNA extracted from mosquitoes was treated using DNase (RNase free DNase set, Qiagen Hilden Germany). A nanodrop spectrophotometer (Nanodrop Technologies UK) and a Bioanalyser (Agilent Technologies UK) were used to assess RNA concentration and quality. After amplification undertaken using 100 ng of total RNA, samples were labelled using Cy-3 or Cy-5 dye with the “Two colors low input Quick Amp labeling kit” (Agilent technologies, Santa Clara, CA, USA). This was immediately followed by samples purification undertaken using Qiagen purification kit. A spectrophotometer (NanoDrop Technologies) and Bioanalyzer (Agilent Technologies) was used to check for cRNA labelling and yield. Labelled cRNAs were hybridized to the ‘*An. gambiae*’ array Agilent 8x15 k chip (AGAM_15 K) (A-MEXP-2196) [46]. After 17 h hybridization at 65 °C and 10 rpm rotation, slides were washed according to the manufacturer instructions (Agilent Technologies). Microarray slides were then scanned with the Agilent G2565 Microarray Scanner System via the Agilent Feature Extraction Software (Agilent Technologies).

Five hybridizations per comparison including three independent biological replicates and two dye swaps were performed. Resistant samples were competitively hybridized against unexposed samples and the Kisumu laboratory strain.

### Microarray data analysis

Genespring GX 11.1 software (Agilent Technologies) was used for microarray data analysis. Comparison of genes expression profiles between groups was undertaken after computing the mean transcription expression ratios to a one sample Student’s *t* test against zero. Benjamin and Hochberg calculation [47] was applied for multiple testing corrections. Transcripts significantly and differentially transcribed were those displaying both *t* test p values <0.05 and a fold change ≥twofold compared to the control or susceptible group. Gene ontology (GO) enrichment was performed using David functional 6.7 [48, 49] to determine GO significantly enriched using as background for comparison the totality of genes differentially transcribed for each group.

### Microarray validation by qRT-PCR (real-time quantitative reverse transcription polymerase chain reaction)

Quantitative RT-PCR analysis as described in Tene et al. [24] was used to confirm the overexpression of detoxification genes detected by microarray. Biological replicates consisting of two micrograms of total RNA per replicate were reverse transcribed into cDNA in a reaction mix containing superscript III (Invitrogen, Carlsbad, CA, USA) and oligo-dT20 primer as recommended by the manufacturer. A MX3005 Agilent system (Agilent) was used to perform quantitative PCR reactions. Each reaction was conducted in a final volume of 25 µl containing iQ SYBR Green supermix (Biorad), primers at the concentration of 0.3 µM each and 5 µl of 1:50 diluted cDNA. The specificity of PCR products generated was verified using melt curves analysis. Standard curves for each gene were generated using serial dilutions of cDNA. Selected transcripts fold changes were normalized to EFGM_ANOGA (AGAP009737_RA) and 40S ribosomal protein S7 (AGAP010592_RA). Fold changes differences of selected genes between test samples and susceptible (Kisumu), were estimated according to the 2^−ΔΔCT^ method considering PCR efficiency [50, 51].

## Results

### Susceptibility to insecticides and species identification

**Fig. 1:**
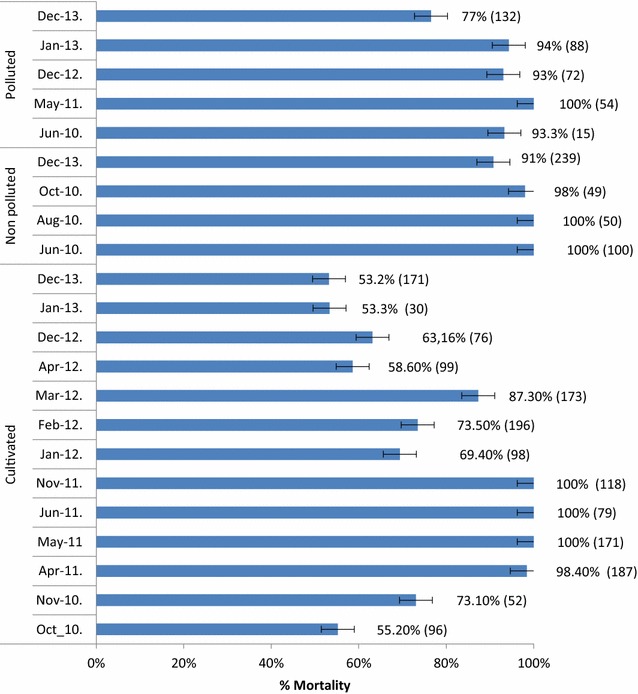
Monthly variation of mosquitoes originating from different breeding habitats susceptibility to bendiocarb in Yaoundé from October 2010 to December 2013; *bars* with standard error

The bendiocarb susceptibility of *An. gambiae* females aged 2–4 days was monitored regularly from October 2010 to December 2013. High variation of mosquito susceptibility according to breeding habitats characteristics was recorded. Mosquitoes originating from cultivated sites were two to five times more resistant to bendiocarb (mortality rate 77.1 %) compared to those originating from polluted (mortality rate 88.4 %) and unpolluted (mortality rate 94.7 %) sites. Mosquitoes originating from polluted sites, also appeared twice more resistant to bendiocarb compared to those originating from unpolluted sites (Table 1). Levels of susceptibility to bendiocarb of mosquitoes originating from cultivated sites apart of 2011 (when a 100 % mortality rate was recorded), were regularly low with mortality rates always below 80 % suggesting an established bendiocarb resistance in this *An. gambiae* population (Fig. 1). High prevalence of DDT, permethrin and deltamethrin resistance was also detected in mosquitoes originating from cultivated sites. However, these mosquitoes appeared highly susceptible to the organophosphate malathion (Table 2). When mosquitoes displaying high bendiocarb resistance (samples collected in 2013) were pre-exposed to PBO before being exposed to bendiocarb, a 100 % (n = 184) mortality rate was recorded. These data suggest the implication of P450 monooxygenase in mosquito resistance to bendiocarb.

**Table 1:** Bendiocarb susceptibility of *Anopheles gambiae s.l.* originating from different type of breeding habitats in the city of Yaoundé

	Cultivated	Polluted	Unpolluted
*Breeding sites characteristics*			
Tested	1428	361	438
Dead	1101	319	415
% mortality	77.1	88.4	94.7

	Cultivated vs polluted	Cultivated vs unpolluted	Polluted vs unpolluted
*Comparison between groups*			
Odds ratio (95 % CI)	2.26 (1.60–3.18)	5.36 (3.46–8.30)	2.37 (1.40–4.03)
p values	<0.0001	<0.0001	0.001

**Table 2:** Mosquitoes from cultivated sites susceptibility to 4 % DDT, 0.75 % permethrin, 0.05 % deltamethrin and 4 % malathion

Insecticides	Nkolondom		Kisumu	
	Tested (dead)	% Mortality (95 % CI)	Tested (dead)	% Mortality (95 % CI)
4 % DDT	274 (15)	5.5 % (3.1–9)	100 (98)	98 % (79.6–119.4)
0.75 % permethrin	138 (13)	9.4 % (5–16)	100 (100)	100 % (81.4–121.6)
0.05 % deltamethrin	161 (92)	57.1 % (46.1–70.1)	100 (100)	100 % (81.4–121.6)
4 % malathion	94 (94)	100 % (86.1–115)	100 (100)	100 % (81.4–121.6)

95 % CI: 95 % Confidence Interval

Of the 233 mosquitoes recorded as resistant to bendiocarb and identified at the species level, 186 (80 %) were *An. gambiae* and 47 *An. coluzzii*. *Anopheles coluzzii* was the predominant species in polluted and unpolluted sites (43/47) whereas *An. gambiae* was the most abundant in cultivated sites (175/186) suggesting an ecological niche partitioning between both species in Yaoundé.

### Screening of ACE-1R mutation

A total of 392 specimens including survivors after exposition to bendiocarb (resistant), dead (susceptible) and control (unexposed) were processed to search for Ace-1^R^ mutation presence. None were detected carrying the Ace-1^R^ mutation. Further supporting the full recovery of susceptibility observed after PBO exposure.

### Genome-wide transcription analysis of bendiocarb resistance

**Fig. 2:**
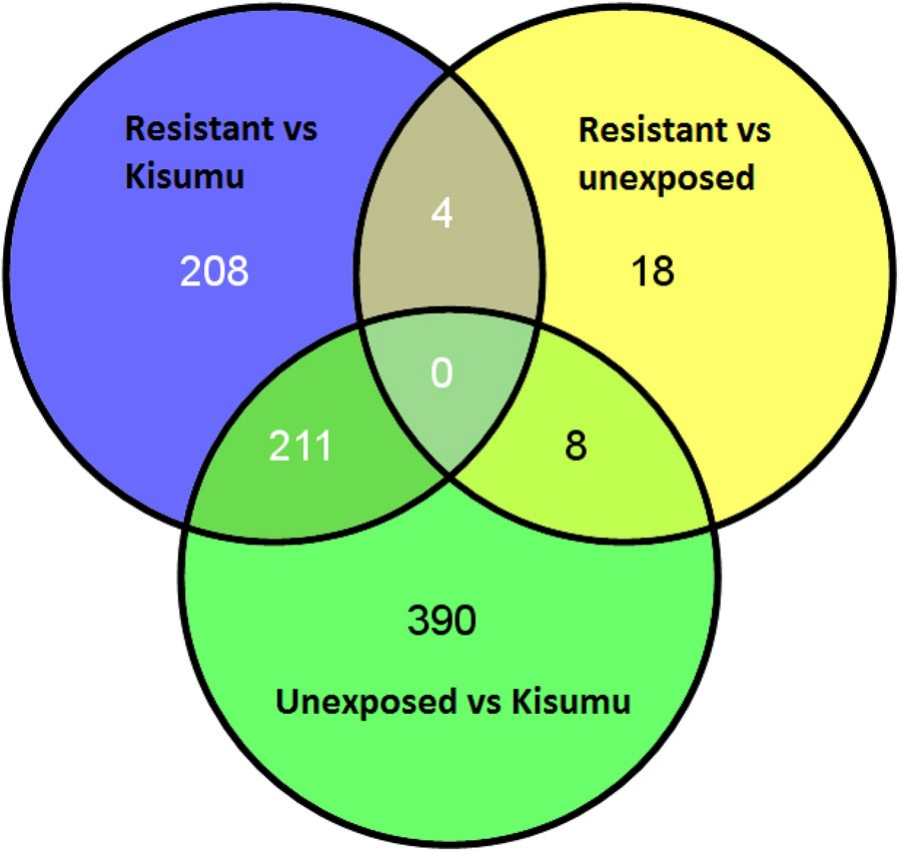
Differentially transcribed genes between resistant, unexposed and susceptible. The *Venn diagram* presents genes with a transcription ratio ≥twofold in either direction and a corrected p value <0.05 in bendiocarb resistant samples compared to unexposed and the Kisumu laboratory strain. Transcripts number are presented for each portion of the* Venn diagram*

Microarray analyses to detect detoxification genes overexpressed, were undertaken with *An. gambiae* samples originating from cultivated sites where mosquitoes display high level resistance to bendiocarb. Three pairwises comparisons were conducted: resistant *vs* control (unexposed) (R_b_-C), resistant vs susceptible (Kisumu) (R_b_-S), control vs susceptible (Kisumu) (C-S). The number of transcripts significantly and differentially transcribed (p < 0.05 and fold-change (FC) >2) varied from 30 between resistant and control (21 up-regulated and nine down-regulated), 423 between resistant and susceptible (Kisumu) (220 up-regulated and 205 down-regulated) and 609 between control (unexposed) and susceptible (Kisumu) (322 up-regulated and 287 down-regulated)(Fig. 2).

### Candidate detoxification genes

A hierarchical analysis was conducted to detect the most likely candidate genes involved in bendiocarb resistance with the assumption that these will likely be detected in more than one comparison. Because no gene was commonly overexpressed in the three comparisons R_b_-C, R_b_-S, C-S, more attention was focused on sets of genes commonly over-expressed between two comparisons.

#### Genes over-expressed in R_b_-S/C-S

The number of detoxification genes commonly over-expressed in R_b_-S/C-S and possibly connected with resistance to bendiocarb, included four cytochrome P450 genes (*cyp6z3, cyp12f2, cyp6m3* and *cyp6m4*) and one Glutathione-S-transferase: gstms3. Four probes belonging to *cyp6z3* gene were detected always over-expressed in R_b_-S and only one *cyp6z3* probe was detected significantly over-expressed in C-S. *cyp6z3* is known to be associated with xenobiotic and insecticide detoxification in *An. gambiae* [52]. Four probes for *cyp12f2* were also found overexpressed with fold changes exceeding 14 in both R_b_-S and C-S comparisons. For *cyp6m3* and *cyp6m4*, the number of probes detected significantly overexpressed varied from one and two for R_b_-S to four and three for C-S comparisons respectively with no important variation of the fold change (Table 3). Both *cyp6m3* and *cyp6m4*, are considered to be involved in xenobiotic detoxification [53]. Genes recorded commonly over-expressed in both R_b_-C and R_b_-S also included three probes for *gstms3*, one probe for each of the three glucosyl glucuronosyl transferases (AGAP005753-RA, AGAP007374-RA, AGAP005750-RA) as well as for xanthine dehydrogenase (Table 3).

**Table 3:** List of genes transcripts displaying the highest over expression fold changes between resistant vs control (unexposed), resistant vs Kisumu (Kis), and control (unexposed) vs Kisumu (Kis)

Systematic name	Description	Fold change		
		Resistant vs control	Resistant vs Kis	Control vs Kis
AGAP008022-RA	cyp12f1	2.29	1.15*	
AGAP009241-RA	cyp4c36	2.12	1.5*	
AGAP001952-RA	cytosol aminopeptidase	5.1	4.1*	1.06*
AGAP009592-RA	zinc carboxypeptidase a1	4.6	−1.92	
AGAP009828-RA	chymotrypsin 1	2.33	1.91*	
AGAP008217-RA	cyp6z3		22.117	16.106
AGAP008020-RA	cyp12f2		19.325	15.801
AGAP005753-RA	glucosyl glucuronosyl transferases		6.174	6.192
AGAP008213-RA	cyp6m3		4.509	2.998
AGAP009946-RA	gstms3		4.378	3.783
AGAP007374-RA	glucosyl glucuronosyl transferases		3.029	2.285
AGAP005750-RA	glucosyl glucuronosyl transferases		6.851	6.228
AGAP008214-RA	cyp6m4		2.622	2.452
AGAP007918-RA	xd24352 Xanthine dehydrogenase		5.31	3.877
AGAP005372-RA	coebe3c		−6.537	−5.798
AGAP008404-RA	glucosyl glucuronosyl transferases		−4.005	−3.136
AGAP002867-RA	cyp6p4		7.237	
AGAP008219-RA	cyp6z1		3.79	
AY745223	cyp6ag1		3.169	
AGAP000165-RA	gstms1		2.578	
AGAP008437-RA	abcc8—abc transporter		2.313	
AGAP004163-RA	gstd7		2.233	
AGAP012308-RA	ornithine decarboxylase		2.18	
AGAP013121-RB	glucosyl glucuronosyl transferases		2.091	
AGAP006725-RA	coeae4 g		−4.438	
AGAP000500-RB	nadph-cytochrome p450 reductase			5.705
AGAP010404-RA	gsts1_1			5.215
AGAP008212-RA	cyp6m2			4.543
AGAP011054-RA	tpx2—thioredoxin dependent peroxidase			3.848
AGAP006222-RA	glucosyl glucuronosyl transferases			3.183
AGAP000163-RA	gstms2			2.095
AGAP013509-RA	carboxylesterase 3			−5.413
AGAP007543-RA	tpx3—thioredoxin dependent peroxidase			−2.57

* Non significant

#### Genes overexpressed in R_b_-C

Further attention was paid to R_b_-C as this compares mosquitoes having similar genetic background and which are only different in the resistance phenotype. Two cytochrome P450 genes *cyp12f1* and *cyp4c36* are over-expressed but with low fold-change of around two including four probes for *cyp12f1* and one for *cyp4c36*. However, the expression level of these two P450 s is low <twofold in R_b_-*S*. Three genes with no recognized role in insecticide detoxification were also over-expressed: Cytosol aminopeptidase, Zinc carboxypeptidase a1 and Chymotrypsin 1 (Table 3).

#### Genes over-expressed only in R_b_-S or C-S

Several cytochrome P450 genes, including four probes for *cyp6z1*, three probes for *cyp6p4*, and three probes for *cyp6ag1* were over-expressed only in R_b_-S. Other genes overexpressed in this comparison included four probes for *gstms1* and *gstd1*-*3*, one probe for *gstd7*, as well as for an ABC transporter and a glucosyl glucuronosyl transferase. For C-S comparison, four probes for *cyp6m2*, three probes for *gstms2*, one probe for *gsts1*-*1* and one for a thioredoxin dependent peroxidase (*tpx2*) were detected overexpressed (Table 3).

### Annotation and gene ontology analysis

Enrichment analysis using DAVID Functional program was conducted to assess GO terms frequent in the group of transcripts up-regulated in resistant vs control (unexposed), resistant or control vs Kisumu. Three GO terms were detected significantly enriched with an enrichment fold of over 20 % when transcripts up-regulated between resistant vs unexposed were analysed. All the terms were associated with proteolysis activity. None of the terms remained significant when the Benjamin and Hochberg multiple testing correction was applied. When the enrichment analysis was conducted with transcripts upregulated between resistant *vs* Kisumu, an enrichment fold varying from 3.2 to 4.5 % was detected for Cytochrome P450 genes (Table 4). Monooxygenase activity remained significant when the Benjamin and Hochberg multiple testing correction was applied (p < 0.01). When transcripts recorded as up-regulated between unexposed vs Kisumu were analysed three were found associated with cytochrome P450 monooxygenase activity with an enrichment fold varying from 2.5 to 2.9 %. However, no activity was scored significantly enriched when the Benjamin and Hochberg multiple testing correction was applied.

**Table 4:** GOTERM categories recorded significantly enriched compare to the reference set (total number of transcripts detected by microarray), terms with a lowest count limit of 2 and an ease score p value <0.05

Category	Go-term functions	FE	p value	Benjamini^a^
*Overexpressed in resistant vs control*				
GOTERM_MF_FAT	Peptidase activity acting on L-amino acid peptides	21	0.009	0.21
GOTERM_MF_FAT	Peptidase activity	21	0.011	0.13
GOTERM_MF_FAT	Proteolysis	21	0.024	0.36
*Overexpressed in control vs Kisumu*				
GOTERM_MF_FAT	Electron carrier activity	5	0.001	0.17
SMART	PhBP	1.7	0.0048	0.25
GOTERM_BP_FAT	Oxidation reduction	5.9	0.0062	0.93
SP_PIR_KEYWORDS	Oxidoreductase	4.2	0.0081	0.46
INTERPRO	Cytochrome P450	2.9	0.0092	0.95
SP_PIR_KEYWORDS	Monooxygenase	2.5	0.012	0.36
INTERPRO	Odorant binding protein PhBP	1.7	0.012	0.85
INTERPRO	Pheromone/general odorant binding protein, PBP/GOBP	2.1	0.013	0.76
INTERPRO	Cytochrome P450, conserved site	2.5	0.019	0.79
*Overexpressed in resistant vs Kisumu*				
GOTERM_MF_FAT	electron carrier activity	7	0.00015	0.02
SP_PIR_KEYWORDS	Iron	5.1	0.00072	0.049
INTERPRO	Cytochrome P450	4.5	0.0011	0.22
GOTERM_MF_FAT	Iron ion binding	6.4	0.0017	0.11
SP_PIR_KEYWORDS	Monooxygenase	3.8	0.0021	0.072
SP_PIR_KEYWORDS	Oxidoreductase	5.7	0.0022	0.051
GOTERM_BP_FAT	Oxidation reduction	7.6	0.003	0.58
INTERPRO	Cytochrome P450	3.8	0.0033	0.3
COG_ONTOLOGY	Posttranslational modification, protein turnover, chaperones	4.5	0.005	0.044
SP_PIR_KEYWORDS	Haem	3.8	0.0058	0.097
GOTERM_MF_FAT	Tetrapyrrole binding	4.5	0.0098	0.36
GOTERM_MF_FAT	Haem binding	4.5	0.0098	0.36

*FE* fold enrichment

^a^Benjamini and Hochberg multiple testing correction

### Validation of microarray data by RT-PCR

**Fig. 3:**
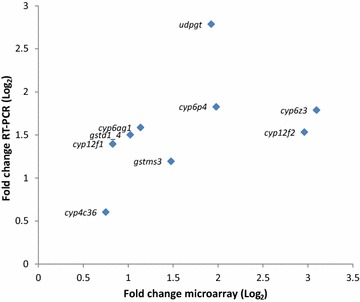
Validation of microarray data by RT-PCR analysis: correlation between microarray data and RT-PCR for nine candidate genes

Eleven transcripts overexpressed in resistant samples including six cytochrome P450 (*cyp6z3, cyp12f2, cyp12f1, cyp4c36, cyp6p4, cyp6ag1),* two GST *(gstd1*-*4, gstsm3)*, two aminopeptidase (cytosol aminopeptidase, chymotrypsin1) and one UDPGT (AGAP005750-RA) were selected to validate microarray data using qRT-PCR. A positive but non-significant correlation (R^2^ = 0.44; p = 0.24) was recorded between qRT-PCR and microarray fold change measurements (Fig. 3).

## Discussion

Despite fast evolution of insecticide resistance in vector populations across Cameroon, molecular mechanisms conferring resistance are still poorly studied. The present study was conducted to characterize molecular mechanisms promoting bendiocarb resistance in *An. gambiae* populations in the city of Yaoundé. Both *An. gambiae* and *An. coluzzii* were found resistant to bendiocarb. Mosquitoes originating from cultivated sites were found to be more resistant to bendiocarb than those collected from polluted or unpolluted sites and could be related to their frequent exposition to xenobiotics including insecticides.

No mosquito was found carrying the G119S mutation conferring target site resistance to carbamate and organophosphate. The increase mortality after the use of piperonyl butoxide (PBO) as synergist suggested the likely implication of cytochrome P450 s in bendiocarb resistance. Our data was similar to previous investigations conducted across West Africa supporting the implication of metabolic mechanisms in carbamate resistance [27]. Although G119S mutation is recognized as the primary resistance mechanism against carbamates and organophosphates it remains less expanded across Central Africa [54]. Its distribution might be constrained by its high fitness cost [39]. However, possession of both G119S mutation and metabolic resistance could lead to extremely resistant phenotypes [27, 40].

Microarray analysis identified several cytochrome P450 genes with the most important being *cyp6z3, cyp6z1, cyp12f2, cyp6p4* and *cyp6ag1,* which were overexpressed when resistant or unexposed samples were compared to the Kisumu susceptible strain (R_b_-S and C-S). However, in addition to their potential implication in insecticide resistance, the high fold change difference detected for some of the genes could likely results from the different genetic background between Kisumu strain originating from Kenya and local *An. gambiae* populations from Cameroon. Similar observations have been reported from previous studies [24]. The over-expression of the two P450 genes *cyp12f1* and *cyp4c36* in the comparison between bendiocarb resistant and control non exposed mosquitoes (R_b_-C) was low and not observed in the R_b_-S comparison suggesting that these genes may not be the main bendiocarb resistance genes. Although further functional characterization studies will help to establish the exact role of these candidate genes. *Cyp12f1* gene was already reported overexpressed in mosquitoes resistant to DDT [53] while no role for *cyp4c36* in insecticide resistance have so far been reported. Nevertheless cytochrome P450 are known to metabolise a large number of xenobiotics including pyrethroids and carbamates [55, 56]. For the set of genes detected only overexpressed in comparison between control and susceptible (C-S), despite a probable absence of role in bendiocarb resistance, it is likely that these detoxification genes (*cyp6m2, gstms2, tpx2, gsts1*-*1*) as well as many others detected over-expressed, might be implicated in the metabolism of an important number of compounds since mosquito populations screened during the study were also recorded resistant to DDT and pyrethroids.

Among potential candidate genes conferring bendiocarb resistance, *cyp12f2* was reported over-expressed in response to bacterial challenge or during malaria parasite invasion in mosquitoes [57] and in permethrin-resistant *An. arabiensis* in South Africa [58]. *cyp6ag1, cyp6z3* and *cyp6p4* were reported over-expressed in DDT and pyrethroid resistant *An. gambiae* and/or *An. arabiensis* populations [53, 58–60]. Ortholog of cyp6p4 and cyp6z3 have been connected to pyrethroid resistance in the malaria vector *Anopheles funestus* [3, 61]. Whereas, cyp6z1 in addition to its confirm involvement in DDT and pyrethroid resistance in *An. gambiae* [62, 63], was recently reported as the main gene conferring metabolic resistance to bendiocarb to *An. funestus* the other major African malaria vector [28].

Previous investigations from Yaoundé identified several candidates genes including *cyp6m2, cyp6p3, cyp6z3, gstd1*-*6*, involved in DDT or pyrethroid resistance [24]. *Cyp6m2* and *cyp6p3* also emerged as main candidate genes conferring bendiocarb resistance in a study conducted in Côte d’Ivoire [27]. However, none of these two genes emerged as potential candidate for bendiocarb resistance. The fact that during the present study only *An. gambiae* individuals were screened for microarray analysis while in Côte d’Ivoire mosquito population screened consisted exclusively of *An. coluzzii* might somewhere explain the difference recorded. Different detoxification gene expression pattern have been recorded for *An. gambiae*, *An. coluzzii* or *An. arabiensis* [53, 59, 64]. Several Glutathione S transferase genes including *gstms3, gstms1, gstd1*-*3* and *gstd7* were also detected overexpressed in R_b_-S and/or C-S comparisons. GSTs are known to metabolize several xenobiotics including pyrethroids, organochlorines and organophosphates and to catalyse the secondary metabolism process of a large number of compounds oxidized by cytochrome P450 [30, 65, 66]. In pyrethroid resistant strains, the overexpression of GSTs attenuates lipid peroxidation induced by pyrethroid and reduce mortality [67].

In the city of Yaoundé, mosquito tolerance to DDT and pyrethroids and the prevalence of the *kdr* allele, have been increasing with time [25, 68]. It remains to be established whether increase resistance to DDT and pyrethroids could also have promoted cross-resistance to carbamates. Yet the increase prevalence of bendiocarb resistance poses serious challenges for malaria control since carbamates are considered as a main alternative to pyrethroids.

## Conclusion

Insecticide resistance is considered as a key challenge for malaria vector control. In this study, we revealed increase tolerance of mosquito to bendiocarb (carbamate). The use of carbamates in IRS are considered as one of the main alternatives to the use of pyrethroid-treated nets particularly in geographical settings with high pyrethroid resistance. Elucidating mechanisms involved in carbamate resistance will enable the monitoring of this resistance in field populations. The data support the implication of cytochrome P450 monooxygenase in mosquito resistance to carbamates however there is a need to conduct further analysis to assess the role of candidate detoxification genes detected during this study.

## Abbreviations

LLINs: long-lasting insecticidal nets
IRS: indoor residual spraying
*Kdr*: knock down resistance
WHO: World Health Organization
PBO: piperonyl butoxide
PCR: polymerase chain reaction
GO: gene ontology
qRT-PCR: real-time quantitative reverse transcription polymerase chain reaction
DDT: dichloro-diphenyl-trichloroethane
